# Madpessimism: A Manifesto

**DOI:** 10.1111/nin.70133

**Published:** 2026-07-02

**Authors:** Simon Adam

**Affiliations:** ^1^ Faculty of Health, School of Nursing York University Toronto Ontario Canada

**Keywords:** abolition, Afropessimism, mad studies, mental health, madness, posthumanism

## Abstract

In this paper, I argue that contemporary psychiatry is confronting a paradigmatic crisis as it collides with posthuman critiques that challenge its foundational assumptions about subjectivity, knowledge, and governance. Rather than a neutral medical practice, psychiatry is theorized as an ontological apparatus embedded within broader regimes of control that classify, regulate, and manage difference through the figure of the Human. Drawing on posthuman philosophy and Afropessimist thought, the article develops the concept of *Madpessimism* to argue that psychiatry's violences are not incidental or reformable, but structurally necessary to a world organized around Humanism's Man—a normative figure that equates rationality, autonomy, and coherence with value and legitimacy. I interrogate emerging developments such as the integration of biomarkers into psychiatric classification, showing how these innovations extend surveillance and deepen the biological inscription of so‐called mental illness. In critiquing reformist calls for “Humanizing” psychiatry, I demonstrate how such efforts often recalibrate rather than dismantle psychiatric power by reproducing the same exclusionary logics that define Humanity itself. Moving beyond genealogical critiques, the analysis reframes psychiatry as a structural condition of possibility for modern governance, rather than a historically contingent institution that can be improved through ethical reform. In developing the idea of Madpessimism, I name a refusal of this framework, rejecting both inclusion within psychiatric systems and the Humanist ontology that sustains them. While refusing the promise of full liberation, this article foregrounds practices of opacity, refusal, and alternative care as modes of survival within conditions of ongoing capture. Ultimately, the paper calls for an abolitionist reorientation that seeks not the reform of psychiatry, but the gradual erosion of its necessity and the Humanist metaphysics that nourish it.

## 1 Introduction

Mounting evidence suggests that psychiatry is entering a paradigmatic crisis as it collides with what Rosi Braidotti (2013, 2019) calls the posthuman condition. What emerges is a struggle between an ideology of control, centralized governance, and categorization, and posthuman frameworks that resist these logics in favor of ontologies of becoming, agential distribution, and entangled human and more‐than‐human worlds (Adam 2026). The evidentiary field surrounding this question is both extensive and heterogeneous, encompassing a wide spectrum of critiques that traverse reformist, reparative, and abolitionist orientations. This body of work does not cohere into a singular analytic position but rather constitutes a dispersed and often internally contested terrain of intervention. Across this landscape, psychiatry is variously problematized as in need of ethical refinement, structural transformation, or wholesale dismantling, reflecting divergent assumptions about the possibility of reform versus the necessity of rupture (Andre 2000; Beresford 2020; Breggin 2008; Burstow 2015; Caplan 1996; Fernando 2017; Healy 2012; Kilty and Dej 2019; Liegghio 2012; Metzl 2011; Modrow 2003; Moncrieff 2009; Pilling 2022; Shim 2021; Szasz 1974; Whitaker 2002).

As psychiatry intensifies its effort to refine a classificatory ontology of sorting and control—most visible in the continual expansion of the DSM—it appears to have reached a threshold at which structural modification becomes necessary for its continued viability, however provisional or potentially futile. In this context, discussions surrounding the forthcoming DSM increasingly point toward a shift away from an exclusively symptom‐based nosology and toward the incorporation of biomarkers in the identification and prediction of so‐called mental illness (Cuthbert et al. 2026; Jha et al. 2025). Within this emerging framework, biomarkers are positioned as measurable biological signals extracted from the body—signals presumed to offer objective insight into mental life. These include technologies that register neural activity, such as electroencephalography, genetic indicators associated with risk or predisposition, and immunological markers, including inflammatory proteins detected through blood analysis. Neuroimaging techniques, particularly functional magnetic resonance imaging, further extend this terrain by mapping neurocircuitry and sorting subjects into purported “cognitive biotypes” that can preconfigure diagnostic interpretation and clinical decision‐making (Cuthbert et al. 2026).

Officially, the DSM continues to present itself as a symptom‐based classificatory system, though the integration of biomarker data subtly reorients psychiatric practice and radically restructures the diagnostic imagination by broadening what counts as clinically perceptible. Biological signals are increasingly mobilized as supplementary tools to refine risk stratification, monitor progress or deterioration over time, anticipate relapse, and justify treatment modification. In doing so, they expand psychiatry's sensory reach beyond clinical observation and reported distress, rendering interior bodily processes newly available for interpretation and intervention. Whatever shape these technologies eventually assume, their introduction reflects not only a restructuring of psychiatric power but also an underlying clinical anxiety within psychiatric ideology. Nonetheless, new modalities of measurement generate new infrastructures of surveillance, extending diagnostic authority into domains previously resistant to capture. As psychiatric governance becomes less reliant on narrative disclosure and so‐called objective clinical observation, more attuned to biological legibility, the mad subject is confronted with an altered terrain of regulation. Far from cultivating transparency or trust, this shift works to more deeply sediment so‐called mental illness in the bodymind of the psychiatrized and the medicalized mad subject. This demands the cultivation of innovative tactics through which mad lives navigate an increasingly technologized regime of assessment and tightening control of diverse forms of information rendered as data that feed the psychiatric oculus.

## 2 Humanization as Recalibration and Capture

Critiques of psychiatric violence are frequently articulated through a reformist appeal to Humanization, some of my own earlier work included (Adam 2017; Hagen 2007; Papadopoulos 2026; van Daalen‐Smith et al. 2019). In this frame, the pursuit of justice is understood as a project of refining psychiatry's ethical sensibilities—developing more nuanced accounts of mental distress and advocating for supports, services, and institutional practices that are perceived as more compassionate, inclusive, and responsive. Humanization, in this sense, is imagined as a corrective intervention: A way of restoring moral legitimacy to an institution that has too often been experienced as coercive or harmful. The political and ethical force of this project derives from a sincere commitment to reconstituting the ontology and subjectivity of the medicalized mad person as fully “equitable” and “inclusive” within existing structures of care. Yet Humanization as a program remains profoundly unstable. It is an ambiguous and contested aspiration that raises unresolved questions about who possesses the authority to confer Humanity, what conditions are assumed to signal its absence, and how one might recognize its successful arrival. If psychiatry is deemed in need of Humanization, what does this imply about its current status? On what basis are we to assume that rendering psychiatry more Humane would necessarily make it less violent, more caring, or more just? The promise of Humanization often exceeds its guarantees, and the problem is compounded by the fact that the very concept of “the Human” is neither neutral nor universal. It is historically constructed, normatively bounded, and anchored to particular ideals of rationality, autonomy, and self‐regulation (Braidotti 2013). Efforts to bring psychiatry into alignment with an acceptably Human standard therefore risk reproducing the exclusions that set that standard in the first place. Rather than resolving psychiatry's violences, Humanization may recalibrate them, reasserting old hierarchies under the banner of ethical progress. These tensions confound the reformist imaginary of the psy enterprise, casting doubt on the assumption that Humanization is either a coherent or sufficient route to justice, much less freedom.

### 2.1 The Problem Is the Hu/Man Himself

Braidotti (2013) critiques the concept of *Man* as not merely a philosophical abstraction but a durable figure of governance that continues to organize Western conceptions of subjectivity and representations of what counts as Human in the modern world. Rooted in Cartesian traditions of rational mastery, moral self‐transparency, and claims to universal Humanity, Man functions as the normative anchor of modern Humanism. Far from being an inclusive or neutral representation of the species, this figure consolidates a highly particular embodiment while disavowing its specificity. Drawing on feminist philosophy, Braidotti foregrounds the symbolic work performed by the Vitruvian Man as an emblem of Humanity. This icon does not simply stand in for the Human as such, but quietly installs a masculinized, heterosexual, European, able‐bodied, rational subject as the measure of the Human Himself. What passes as an abstract ideal is therefore anything but abstract. The Humanness condensed in this figure presumes a subject who is cognitively coherent, morally self‐governing, physically ordered, and implicitly entitled to universality. The distance between this ideal and the lived conditions of most lives is substantial, yet it is precisely this gap that enables the figure of Man to operate as a Cartesian sorting device—distinguishing full Humanity from its partial, deficient, or failed approximations. Braidotti's intervention exposes how the symbolic neutrality of Man conceals a norm that must continually exclude in order to stabilize itself.

Despite sustained critique, modern subjectivity remains deeply entangled with this Humanist architecture. The Cartesian subject—self‐knowing, autonomous, and morally accountable—continues to reappear within contemporary regimes of psychiatry and associated governance, particularly under neoliberal imperatives of self‐management, productivity, optimization, and risk. Autonomy becomes obligation, transparency becomes virtue, and self‐regulation becomes the condition of recognition. In this way, Man endures not as a philosophical relic, but as a living standard through which subjects are evaluated, disciplined, and rendered intelligible. Once Man solidifies into the dominant figure through which Humanity is defined, He ceases to be merely representative and instead becomes a sorting mechanism. He names the Human while simultaneously cleaving it, producing difference through acts of distinction that endlessly restage the hierarchies He claims to overcome. Man operates as a paradoxical totality: An abstract universal that can only stabilize itself by dividing, ranking, and managing. His endurance depends on the continuous production of ontological others as deficit—figures constituted as incomplete or improper in relation to His form. What appears as unity is thus achieved only through regulated separation. Through this process, Humanness is carved into ordered fields—types, ranks, classifications, and normative genres—out of which the infrastructures of governance, discipline, and sanctioned harm are built. In psychiatric knowledge, this Humanist Man appears as both unmarked and exemplary. He is at once presented as simply ordinary and quietly elevated as the standard to which all others must answer. This oscillation grants Him absolute epistemic authority over those whose lives hover at the edge of intelligibility. As the embodiment of “normal life,” He becomes the benchmark against which difference is measured and judged, defining the traits required for recognition as fully Human, traits that closely resemble Man Himself. Drawing from Braidotti's critique, these traits emphasize whiteness, maleness, heterosexuality, cognitive mastery, emotional consistency, and bodily orderliness. Crucially, such traits are not exceptional because they are widespread, but because they describe a socially dominant minority—what Deleuze and Guattari (1987) identify as the majoritarian position. Masculinity, whiteness, able‐bodiedness, rationality, and related marks of dominance underpin Man's global authority, shaping the normative assumptions that organize psychiatric reason.

Within clinical practice, these traits function as an unspoken baseline. Difference from this baseline is rendered pathological, giving rise to structural racism, sexism, ableism, sanism, and allied violences. The idealized bodymind that Man represents becomes the silent measure of value, against which all other forms of life appear aberrant. In this configuration, desirability and exception collapse into a single horizon of the Human. Man becomes the tacit template through which diagnosis, judgment, and intervention proceed. Those who do not conform—the racialized, disabled, neurodivergent, feminized, and irrational—are positioned as His necessary outside: Denied full existential legitimacy even as they are made legible through classificatory regimes. Man thus installs Himself as Humanity's universal proxy while authoring the margins that secure His dominance.

## 3 Psychiatry Not as Accident but as Architecture

The violences associated with psychiatry are most often narrated as deviations from an otherwise would‐be‐Humane project. Institutional harm is framed as error, excess, or failure: The misapplication of a fundamentally therapeutic mandate, the corruption of care by stigma, racism, underfunding, or professional misconduct. Within this dominant interpretive frame, coercion is understood as an iatrogenic, regrettable malfunction rather than as a constitutive feature. Psychiatry, we are told, means well—even when it confines, medicates against refusal, surveils intimacy, or suspends civil status. Harm appears as interruption, yet care remains the presumed core.

This narrative structure performs crucial political work. By locating violence in contingency rather than design, it protects psychiatry's legitimacy while rendering abuses legible as correctable. If harms result from bias, austerity, or insufficient training, then the solution is reform: Better oversight, enhanced education, improved ethics, more inclusive vocabularies, expanded participation, and so on. Psychiatry thus persists as an institution perpetually in need of repair but never of abolition. Yet this interpretive habit profoundly misunderstands psychiatry's role in the ordering of the modern world. The harms psychiatry produces are not accidental residues left behind by an otherwise benevolent practice. They are not the price paid for imperfect implementation. They are the ordinary effects of an apparatus whose coherence depends upon the management of lives deemed excessive, unintelligible, or unsafe (Burstow 2015; Szasz 1974). Psychiatry does not occasionally fail care but organizes it through failure—by stabilizing normativity through the identification, regulation, and containment of those who disrupt it.

To reckon seriously with psychiatry therefore requires a shift in register. Policy critique and ethical reform, while not irrelevant, operate downstream of a more fundamental problem. While policy critique and rights‐based reform have, for decades, sought to contest psychiatric power, these efforts have paradoxically coincided with psychiatry's continued expansion and intensification. Rather than diminishing its authority, contemporary psychiatry has increasingly consolidated its epistemic and institutional reach through more individualized and biologized frameworks of distress—exemplified, for instance, by the growing prominence of biomarker discourse and neurobiological paradigms of diagnosis. What must be interrogated is not psychiatry's methods but its ontology and that of the Human that sustains its immunized perpetuity. Psychiatry does not merely respond to madness. It presupposes a world in which madness already exists as a problem to be solved. That world is structured by a particular figure of the Human I describe above.

Learning from Posthuman (Braidotti 2013, 2019) and Afropessimist (Warren 2018; Wilderson 2021) critiques of Humanism's Man and His calculus of classification, psychiatry can be reframed as one of the institutional relays through which this ontological sorting is enacted. It supplies the language, protocols, diagnoses, and expert rationales that translate metaphysical judgments into actionable force. Under the sign of care, psychiatry naturalizes the idea that certain minds require oversight, correction, or containment. Distress is individualized, structural abandonment is psychologized, relational rupture is internalized as pathology, and madness becomes evidence of personal failure rather than a sign of social unlivability and/or world‐making collapse. Intervention thus appears as necessary and benevolent even when it arrives in the form of confinement, sedation, surveillance, or juridical suspension. Violence is rendered therapeutic by being routed through expertise, and the authority to help becomes the authority to harm.

Within this architecture, as long as psychiatry remains tethered to Man's grammar—its equivalence of reason with worth, autonomy with legitimacy, compliance with safety—efforts to reform it will reproduce the same violences in altered form. The survivor will have been consulted: New vocabularies will emerge. Training modules will proliferate. Ethics statements will circulate. But the underlying distribution of vulnerability will remain intact. What is required is not only better practice in the meantime, while other options are lacking within the existing frame, but an eventual rupture with the frame itself. This rupture is what I name *Madpessimism*.

### 3.1 At the Limits of Genealogy

Michel Foucault (1988) demonstrates that psychiatry does not simply discover mental illness but produces madness as an object through regimes of knowledge, normalization, and care. His work exposes how coercion is rendered benevolent, how expertise authorizes violence, and how deviation is disciplined through therapeutic means (Foucault 1995). Yet Foucault ultimately treats psychiatry as a historically contingent formation—an apparatus that emerges under specific social conditions and therefore remains, at least in principle, transformable. Madpessimism departs at this point. It argues that psychiatry is not merely a historical configuration of power, but an ontological requirement of a world organized around Man. Much like anti‐Black racism, as Afropessimists contend, is needed in order to justify the completeness of the white subject and its ontological superiority, psychiatry is a necessary apparatus that justifies the sanity of Man and His sanist ontology. Where Foucault discursively traces how madness is governed, Madpessimism asks why the world requires madness to be governed at all. This shift relocates critique from genealogy to structure. Psychiatry is not a failed or corrupted institution awaiting repair, but a necessary support beam of a regime that equates reason with worth, autonomy with legitimacy, and compliance with safety. In this sense, Madpessimism refuses the Foucauldian horizon of counter‐knowledge. It insists that as long as the Man of Humanism remains a normative figure, psychiatry will remain indispensable. The problem is not the mismanagement of madness, but the world that renders madness governable in the first place. Madpessimism refuses the consoling belief that psychiatry has merely lost its way. It begins from the recognition that psychiatry functions as architecture: A structuring technology of the Human, not a service that intermittently malfunctions. Its task is not improvement but exposure—making visible the conditions under which madness becomes governable, legible, and disposable.

## 4 A Madpessimist Ontology

Madpessimism draws its methodological clarity from Afropessimist thought, namely drawing from the work of Calvin Warren (2018) and Frank B. Wilderson III (2021), particularly its insistence that some forms of violence are not problems to be solved but conditions to be confronted. Afropessimism argues that anti‐Blackness is not a correctable injustice within an otherwise just system. It is the organizing logic of the modern world itself. The world coheres through the ongoing production of Blackness as social death. Freedom, within this framework, cannot be achieved through inclusion, representation, or reform. It names a limit: A recognition that the structures that require anti‐Blackness cannot be Humanized from within. This insight sharpens how psychiatry must be understood. If the world is structured by anti‐Blackness, then the disciplinary formations that emerge within it—including psychiatry—cannot be neutral technologies gone astray. They are downstream expressions of a deeper ontological order. Psychiatry's authority to name, diagnose, and intervene depends upon a background assumption that some lives are already misaligned with the world's normative form. Madness does not enter psychiatry as an open question but arrives as a predesignated problem.

Within this landscape, the figure psychiatry governs—the medicalized mad subject, as Bruce (2021) puts it—occupies a structurally paradoxical position. This subject is indispensable to psychiatry's legitimacy—without madness, psychiatry would have no mandate—yet is never granted full entry into the domain of valued personhood. The medicalized mad subject is held at a permanent threshold: Conditionally included, endlessly monitored, and perpetually corrigible. Distress must be translated into symptoms, and lived narratives must be rendered clinically legible. Psychiatric space thus operates less as a site of healing than of sorting, where some lives are stabilized and protected while others remain indefinitely triaged. Some are framed as salvageable, while others are managed as risks. This is not the outcome of scarce resources or flawed professionals. It is the mechanism through which psychiatry reproduces its authority. The institution's power lies precisely in its capacity to discriminate—to decide who can be ostensibly rehabilitated and who must be contained. Calls to “restore Humanity” to psychiatric practice falter here, because Humanity itself—under Man's regime—is the gate through which exclusion operates. To be recognized as Human is to meet Man's criteria. These are not neutral capacities but moral technologies that sort those who deserve protection from those who remain exposed, surveilled, and managed.

Human rights discourse illustrates this bind with particular clarity. When mobilized in psychiatric contexts, rights do not function as unconditional guarantees. They operate as tests. One qualifies for rights by performing rational competence, risk awareness, and insight into one's condition. Rights thus become contingent achievements rather than baseline entitlements. Those who cannot—or will not—perform the required norms remain subject to intervention, confinement, or abandonment. Madpessimism refuses the hope that this structure can be reformed into justice. It does not seek better representation of mad subjects within psychiatric governance and its adjacent medico‐legal structures, nor a more compassionate diagnostic language. It likewise rejects the figure of Man as gatekeeper of so‐called Humanity, along with the politics of recognition that seek the integration of the mad subject into His moral order. It names what cannot be repaired. Like Afropessimism, it insists that the problem is not exclusion to be remedied by way of restoring rights to a marginalized Human but the Human itself as a regulatory fiction. The task, then, is not inclusion but refusal.

### 4.1 The End of the World as Analytic Horizon

Afropessimism famously argues that nothing short of the end of the world can satisfy the conditions of Black freedom. This claim is often misunderstood as apocalyptic fantasy or nihilistic despair. In fact, it is a rigorously analytic proposition. If the world is constituted through anti‐Blackness, then Black freedom cannot arrive without the dissolution of that world. Afropessimism confronts the violent teleology through which the thriving of the modern world is contingent on the simultaneous construction and annihilation of the Black subject. Reform, recognition, and inclusion cannot resolve an ontological structure designed to require Black social death. What is required is another world, a postmetaphysical otherwise. Madpessimism advances a parallel claim with respect to psychiatry. The freedom of the mad subject cannot be accomplished through incremental reform, participatory inclusion, or diagnostic refinement. It requires the end of the world that renders madness intelligible as deviation in the first place. To speak of the end of the world here is not to celebrate destruction but to name a point of refusal: A recognition that the foundational assumptions organizing care, safety, and value are themselves the problem.

This framing aligns with Wilderson's (2021) abolitionist method while also marking an important divergence. Wilderson positions Blackness as occupying a singular relation to social death—outside civil society in a way other marginalized groups are not. Madness, disability, queerness, and other forms of exclusion are often described, within this framework, as holding “junior partnerships” within the anti‐Black world while benefiting from anti‐Black violence. While I share Wilderson's insistence on the structural centrality of anti‐Blackness, I approach the figure of Man somewhat differently. Man's operations are ruthless but flexible. Vulnerability to exclusion is not determined by a single axis of identity but by proximity to normativity at a given moment. Recognition is unstable. It must be continually earned and can be abruptly withdrawn. Psychiatry exploits this instability, governing through conditional shelter. From this perspective, no marginalized group occupies a permanently fixed position relative to Man. Subjects and communities are compelled to negotiate proximity to normativity in order to survive. Relating to this, Frantz Fanon (2008) asks: “Is there in truth any difference between one racism and another? Do not all of them show the same collapse, the same bankruptcy of man?” (p. 86).

The example of a straight Black man distancing himself from a visibly queer or feminized man in a public space clarifies this dynamic. Such an act does not transform the Black man into a beneficiary of anti‐queerness. It reflects a tactical calculation within a world that distributes danger unevenly but relentlessly. Given that proximity can increase vulnerability, distance can sometimes mitigate harm. These negotiations—often misread as complicity—are better understood as strategic engagements with exposure. They reveal how Man governs through scarcity and threat, offering limited protection to those who can approximate His norms while keeping all others in precarity.

Psychiatry functions as a critical site where parallel negotiations are formalized. It offers recognition in exchange for compliance, stability in exchange for legibility, and safety in exchange for surrender. Inclusion within psychiatric systems does not abolish the criteria by which exclusion operates but reshuffles access to them. This is why reforms that promise inclusion so often disappoint, because to be included within Man's world is to remain subject to His adjudication. Madpessimism names this impasse without seeking escape through optimism. It refuses the fantasy that psychiatry can be rendered benign or that Humanity can be expanded without reinstalling its gate. The end of the world here names not annihilation but analytic clarity: A commitment to stop mistaking cruelty for care and escape for freedom.

### 4.2 Refusal, Composition, and Life After Man

The “pessimism” in Madpessimism can be misread as despair. However, it is more productively understood as analytic refusal. It names a commitment that emerges when psychiatry is recognized not as a flawed service but as ontological technology. To argue that exiting psychiatry requires the end of the Human is not to indulge in hyperbole. It is to acknowledge that as long as Man remains the measure of value, madness will remain governable, permanently occupying the status of semihumanity. Refusing Man means refusing a system that offers safety only through surveillance and care only through compliance. This refusal does not target persons. It targets the metaphysical arrangements that decide who counts as one. What becomes possible after refusal is not utopia but reorientation. When rationality is no longer treated as the metric of value, the demand that distress be neatly articulated and administrable loses its force. Care can be reimagined not as correction but as sustenance. The horizon shifts from individualized compliance to collective sufficiency. This shift opens space for what I refer to as *ecologies of care*—configurations of support that do not require diagnostic translation, professional legitimation, or psychiatric authorization. Housing, income, food, companionship, and meaning‐making can be organized as baseline supports rather than therapeutic rewards. So‐called crisis can be met with sanctuary rather than force. Altered states can be held without immediate recourse to interpretation or control, despite their “worrisome” manifestations.

Madpessimism also guards against two persistent, dualist errors: Pathologization and romanticization. Madness cannot be reduced to pathology nor contained as spectacle. It unfolds as a shifting mode of being—at times painful, at times generative, often both, and at other moments eluding interpretation and representation altogether. The task is not to cure madness or aestheticize it, but to detach it from the apparatus that claims ownership over it—whether medical or political. Abolition, in this frame, is not merely the dismantling of institutions. It is a compositional practice that involves eroding the predicates that authorize psychiatric power and its signifying metaphysics, while building infrastructures that meet needs differently. Abolition is not the absence of care. It is care organized outside Man's jurisdiction, post‐psychiatry and eventually, post‐madness.

To reiterate, Madpessimism remains indebted to Black study for its rigor about limits. Anti‐Blackness is not a metaphor or a policy failure, but the ground of the modern world. Madpessimism neither seeks to collapse madness into Blackness nor claim equivalence. It learns, instead, how to identify what cannot be fixed. From this ground, Madpessimism rejects identity when it becomes a passport to conditional inclusion or a liability to the subject's exposure to the psy sensorium. Participatory capture—where mad subjects are invited into governance structures that leave psychiatric power intact—must be named as such. Thus, postidentitarian madness (Adam 2026) prioritizes material reductions in exposure to psychiatric force over representational parity. It builds coalitions oriented toward survival rather than recognition.

If Madpessimism names what must end, it also gestures toward how life persists in the meantime. Escape is not a destination but a method, unfolding through study, refusal, the withdrawal of legitimacy, and the slow invention of alternatives. This work is precarious and incomplete. It does not imagine a final victory but aims instead to make psychiatry obsolete by making it unnecessary, implausible, and finally unthinkable. Ending Man is not the end of us. It is the end of a world in which life must be adjudicated before it can be supported. It names the condition under which exiting psychiatry becomes not an act of defiance, but the ordinary texture of living together.

A Madpessimist praxis maintains unsentimental expectations of freedom and does not claim to possess a key that might release the mad subject once and for all. Instead, it situates itself in an extended horizon of attrition, as Burstow (2014) articulated, attending to the slow erosion of psychiatry and its interlocking architectures of violence while remaining oriented toward the possibility of an affirmative post‐mad futurity. This posture is not one of passive endurance. It is an active engagement in the materialities of abolition—an attunement to moments of fracture, openings, and instability within the psy order. From these sites, Madpessimism activates lines of flight and provisional escapes, fully aware that such movements are fragile, reversible, and frequently reterritorialized.

## 5 What Does This Mean for Nursing?

This argument carries significant implications for the nursing discipline. Where nursing remains invested in the reform of the psy apparatus, it must relinquish the belief that such reform is viable. Psychiatry is not a system that has merely lost its ethical footing or requires technical correction. It is structurally irredeemable (Burstow 2015). Efforts to “improve” psychiatric practice through reformist interventions—however well intentioned—do not weaken the institution. Instead, they risk expanding its reach, refining its legitimacy, and increasing its capacity for capture. Reform does not interrupt psychiatric power. It optimizes it. Recognizing this requires a redirection of nursing's political, ethical, and material energies. Practices commonly framed as progressive—patient consultation, the integration of lived experience, and policy reform—operate largely within psychiatry's governing logics. While these efforts may improve individual encounters, they ultimately tinker with an apparatus whose core function remains intact. Worse, they risk being absorbed by the institution itself, transforming critique and resistance into new forms of professional expertise that reinforce psychiatric authority. Nursing must therefore confront the uncomfortable reality that reformist labor often stabilizes the very system it seeks to soften.

This does not entail withdrawal from care, advocacy, or engagement in the present. On the contrary, nursing must prioritize work grounded in the immediate material conditions of subjects signified as mad. This includes sustained advocacy around housing, income security, employment, access to education, food, and other social determinants of health, and such work is already underway in many contexts. The critical shift proposed here is not one of action, but of orientation. Nursing must decouple this material advocacy from the expectation that it will culminate in meaningful transformation of the psy institution itself. These interventions mitigate harm, but they do not dismantle the structure that produces it, given its ontological ground remains stable as long as Man continues to govern. Accepting this claim does not require resignation. Instead, it calls for a more sustainable approach to struggle—one that attends to the material conditions of the present while sustaining a critical commitment to a postmetaphysical, abolitionist future. Accordingly, while nursing participates in important and necessary day‐to‐day advocacy and activist efforts alongside mad communities, it must refuse the consoling illusion that such work will ultimately liberate mad subjects from psychiatric violence or from the Humanist norms that authorize that violence. Relief must not be mistaken for resolution. Equally urgent is the demand that nursing, in the present moment, refuse participation in the violent, disciplinary, and carceral practices that continue to operate under the name of care. This might take shape in nurses' redirecting of patients and communities away from psychiatric capture and toward alternative care pathways and non‐pathologizing affirmative supports (Ford‐Jones et al. 2026). This is no small demand. Nursing's deep entanglement within the psy system affords it a degree of institutional power, but one that also secures its immobility. That power binds nursing to the very structures it might otherwise resist, a paradox that constrains its capacity for refusal and forecloses more radical forms of ethical and political action.

Future‐oriented nursing practice must cultivate imaginaries grounded in a philosophy of care that is unyieldingly abolitionist—both institutional and metaphysical. This requires holding open the necessity of psychiatry's abolition—alongside its Humanist gatekeeping figure of Man—while continuing to provide care and intervention in the immediate conditions of mad life. The task is not to await a future beyond psychiatry before acting, but to practice care in ways that are no longer anchored to the institution's promise of repair or legitimacy, even if it is risky. Nursing must therefore learn to work in a tension between the present and the unthinkable: Intervening without reproducing the structures it ultimately seeks to undo. The long‐term horizon of this project is for nursing to reconceptualize itself not as a stable professional authority, but as a postdisciplinary effect of the care needs articulated by mad communities themselves—one contingent and temporary resource among many. In this framing, the discipline exists at the community's disposal—answerable to collective survival rather than institutional and professional identity and disciplinary continuity. Such a shift demands that nursing imagine itself as philosophically mobile and processual, capable of evolving alongside the gradual attrition of psychiatry and the violent Humanist assumptions that underwrite it. Rather than securing its future through professional preservation, mental health nursing must remain open to transformation, contraction, or even disappearance. Its role in caring for mad subjects may radically change, fragment, or become obsolete as alternative epistemologies and infrastructures of care take root. This is not a failure of the discipline but a measure of its ethical seriousness. In a postmetaphysical world, nursing's value will lie not in preservation and permanence, but in its capacity to relinquish authority in favor of responsive, collective care.

## 6 Conclusion

In this article, I have argued that psychiatry's contemporary crisis cannot be adequately understood as a problem of outdated methods, ethical drift, or insufficient reform. Rather, psychiatry encounters sustained resistance because it remains tethered to a Humanist ontology increasingly incompatible with the posthuman condition (Braidotti 2013). Despite gestures toward innovation—whether through diagnostic expansion, appeals to Humanization, or the incorporation of biomarkers—psychiatry continues to organize itself around a figure of the Human that demands coherence, autonomy, rationality, and self‐regulation as the price of recognition. These demands are not incidental distortions of an otherwise benevolent institution but the very grammar through which psychiatric power operates. The turn to biomarkers and biological legibility illustrates this impasse with particular clarity. While often framed as progress or objectivity, these technologies extend psychiatry's reach into ever more intimate domains of life, deepening rather than disrupting its capacity for surveillance, sorting, and control. Likewise, reformist appeals to Humanization, however well intentioned, fail to escape the logic they seek to undo. This is because psychiatry *is* and *has always been* Humane—it is a Humane apparatus par excellence. By presuming that violence results from psychiatry's failure to adequately recognize Humanity, reform surreptitiously reproduces the authority of a figure of Man whose criteria for Humanness are themselves exclusionary. Humanization thus recalibrates psychiatric power without undoing its conditions of possibility, while masquerading as social progress.

Against this backdrop, Madpessimism names a necessary rupture. It departs from genealogical critiques that locate psychiatry's violences in historical contingency alone, and instead insists that psychiatry functions as architecture: A structuring technology of a world organized around Man. As long as this figure remains intact, psychiatry will remain a structural requirement for modern society, regardless of how refined or “compassionate” its practices become. Importantly, Madpessimism does not confuse refusal with resignation, because its pessimism is analytic rather than nihilistic. It rejects the fantasy of repair while remaining attentive to how life persists within, against, and beyond psychiatric capture. Escape is understood not as a final liberation but as a method—fragmentary, reversible, and continuously contested. From this orientation, practices of refusal, opacity, and composition can take shape, working to unsettle psychiatric authority by interrupting legibility while cultivating forms of care that do not depend on diagnostic validation or recognition within Humanist frameworks. In this sense, Madpessimism does not promise freedom as a destination. It offers, instead, a disciplined clarity about limits and a commitment to making psychiatry increasingly irrelevant, implausible, and finally unthinkable. What ends here is not care, solidarity, or collective responsibility, but a metaphysical world in which life must be adjudicated before it can be supported. If there is an affirmative horizon in Madpessimism, it lies not in the Humanization of psychiatry, but in the slow invention of forms of living that no longer require psychiatry and its reigning figure of Man to endure.

## Ethics Statement

The author has nothing to report.

## Conflicts of Interest

The author declares no conflicts of interest.

## Disclosing the Use of Artificial Intelligence

The author declares the use of Microsoft 365 Copilot software version 1.2604.2102.02 for language polishing, conciseness, and formatting purposes (according to Wiley policies, no disclosure is necessary).

## Data Availability

Data sharing not applicable to this article as no datasets were generated or analyzed during the current study.
